# Bioactive antibacterial silica-based nanocomposites hydrogel scaffolds with high angiogenesis for promoting diabetic wound healing and skin repair

**DOI:** 10.7150/thno.41839

**Published:** 2020-03-31

**Authors:** Yannan Li, Tianzhen Xu, Zhuolong Tu, Wentong Dai, Yumeng Xue, Chengxuan Tang, Weiyang Gao, Cong Mao, Bo Lei, Cai Lin

**Affiliations:** 1 Department of Burn, the First Affiliated Hospital of Wenzhou Medical University, Wenzhou 325000, China; 2 Key Laboratory of Orthopedics of Zhejiang Province, the Second Affiliated Hospital and Yuying Children Hospital of Wenzhou Medical University, Wenzhou 325027, China; 3 School of Physical Science and Technology, Inner Mongolia University, Hohhot 010021,China; 4 Frontier Institute of Science and Technology, Xi'an Jiaotong University, Xi'an 710054, China; 5 Department of Orthopedics, Zhuji People's Hospital of Zhejiang Province, Shaoxing 312000, China

**Keywords:** silica-based biomaterials, bioactive scaffolds, multifunctional properties, diabetic wound healing, tissue engineering

## Abstract

Diabetic wound repair and skin regeneration remains a worldwide challenge due to the impaired functionality of re-vascularization.

**Methods**: This study reports a bioactive self-healing antibacterial injectable dual-network silica-based nanocomposite hydrogel scaffolds that can significantly enhance the diabetic wound healing/skin tissue formation through promoting early angiogenesis without adding any bioactive factors. The nanocomposite scaffold comprises a main network of polyethylene glycol diacrylate (PEGDA) forming scaffolds, with an auxiliary dynamic network formed between bioactive glass nanoparticles containing copper (BGNC) and sodium alginate (ALG) (PABC scaffolds).

**Results:** PABC scaffolds exhibit the biomimetic elastomeric mechanical properties, excellent injectabilities, self-healing behavior, as well as the robust broad-spectrum antibacterial activity. Importantly, PABC hydrogel significantly promoted the viability, proliferation and angiogenic ability of endothelial progenitor cells (EPCs) *in vitro*. *In vivo*, PABC hydrogel could efficiently restore blood vessels networks through enhancing HIF-1α/VEGF expression and collagen matrix deposition in the full-thickness diabetic wound, and significantly accelerate wound healing and skin tissue regeneration.

**Conclusion:** The prominent multifunctional properties and angiogenic capacity of PABC hydrogel scaffolds enable their promising applications in angiogenesis-related regenerative medicine.

## Introduction

Chronic diseases such as autoimmune diseases, diabetes and chronic skin trauma, are still difficult to cure and the medical need is still far from satisfactory 1. As one of the most common chronic disease, the skin wound in diabetes is difficult to be healed completely, due to their low blood flow supply, poor neutrophil antimicrobial ability and disordered inflammatory response 2. As a typical common diabetic complication, more than 750,000 cases of diabetic foot ulcer (DFU) emerge in America every year, about 10% of which require amputation of lower limbs 3. Various strategies have been used to treat and promote the diabetic wound healing 4. As the promising treatment methods, the cellular and growth factors-based therapies have shown good results, but they are usually costly and difficult to clinic translation 5,6. Therefore, the development of highly bioactive biomaterials-based wound dressing has become urgent and hot topic in treating chronic injury such as diabetic wound 7.

Up to know, various biomaterials dressings, including porous matrix, transparent film, ointment/powder and biomedical hydrogel, have been employed in clinic 8. Additionally, to give the favorable microenvironment for wound healing, multifunctional biomaterials dressings are also developed, such as antibacterial activity, hemostasis, antioxidant ability 9-11. Specially, hydrogel-based biomaterials have the similar physical structure with natural extracellular matrix (ECM) and have shown promising results in improving wound healing 12-16. However, most of reported biomaterials dressings showed the absence of bioactivity that could significantly enhance the early angiogenesis and induce skin tissue formation. Recently, protein/peptide/exosome-functionalized biomaterials showed excellent bioactivity in enhancing diabetic wound healing and skin regeneration 17-22. Our group also developed an exosome-based self-healing hydrogel which showed the enhanced effect on chronic diabetic wound healing and complete skin regeneration 23. However, these reported bioactive dressings were mostly still dependent on the activity of biologics but not the biomaterials themselves.

Bioactive glasses (BGs) have shown very special biological properties including osteogenic ability, bone/soft tissue bonding activity and promoting angiogenesis 24-28. Bioactive glass nanoparticles (BGNs) have also exhibited several promising biomedical applications in bone regeneration, drug and gene delivery and bioimaging 29-32. Our previous studies also indicated that BGN could enhance the blood vessels formation in diabetic wound healing through activating the HIF-1α/VEGF signaling pathway 33,34. On the other hand, copper (Cu) is an essential trace element for humans and affects the wound healing process including angiogenesis, expression and stabilization of extracellular skin proteins, normal melanin formation and maintenance of normal hair structures 35,36. Simultaneously, copper ion (Cu^2+^) has excellent antibacterial properties, which can reduce the possibility of wound infection to promote wound healing 37. However, the increased non-physiological concentrations of Cu^2+^ also increase the risk of ion poisoning. Therefore, the controlled release of Cu^2+^ could efficiently decrease the cytotoxicity and improve their biological activity. It is very interesting and worthy to integrate the monodispersed BGN containing copper (BGNC) into a biocompatible macromolecule network to create a bioactive self-healing antibacterial dressing for enhancing diabetic wound healing.

**Scheme 1:**
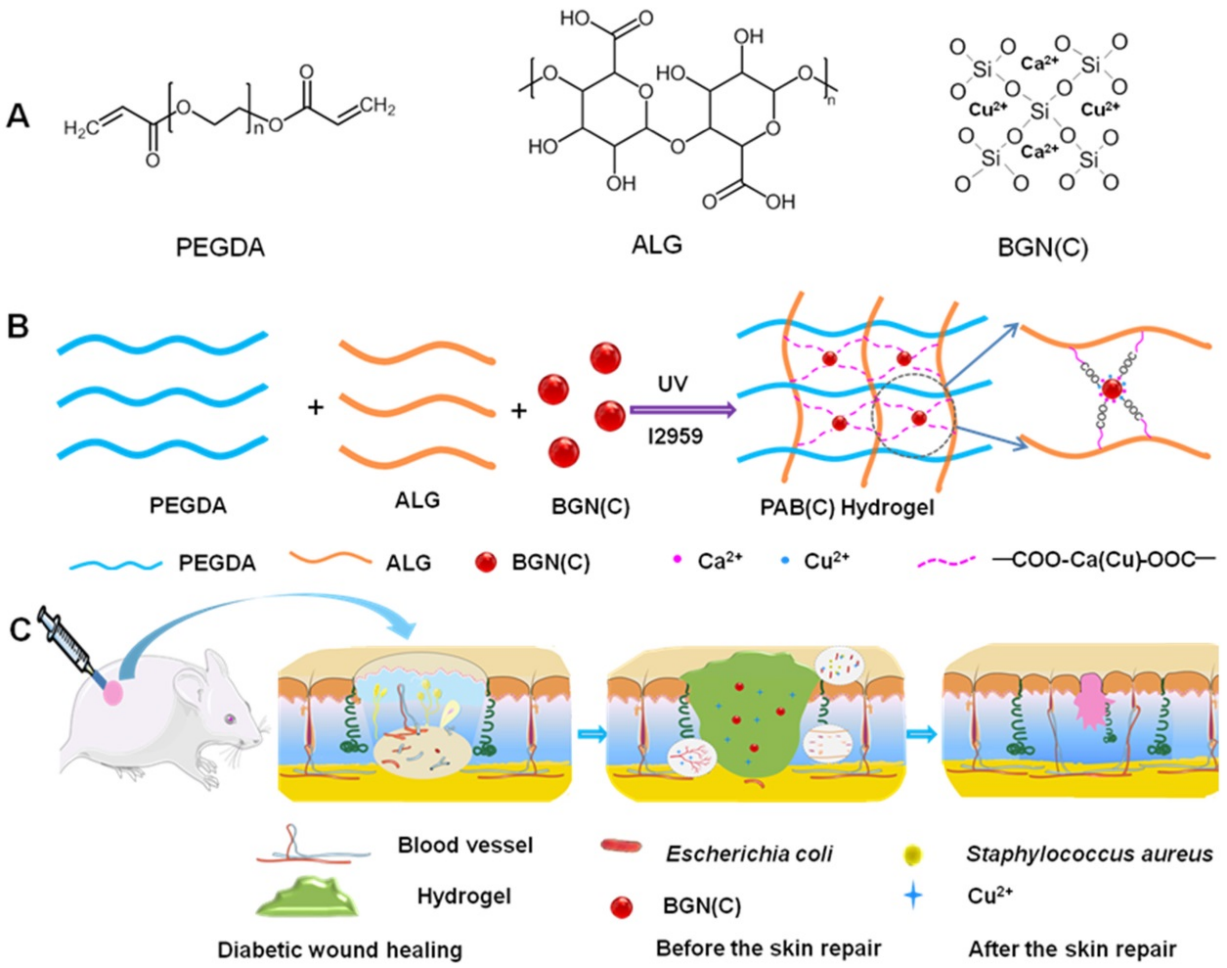
** Synthesis and potential wound healing application of PABC hydrogel. (A)** Main components of PABC hydrogel including PEGDA, ALG and BGN; **(B)** Schematic representation of PABC hydrogel formation; **(C)** Potential application in diabetic wound healing and hypothetical mechanism.

Herein, without adding any biologics or drugs, we report a complete biomaterial dressing with robust antibacterial activity and self-healing ability, which would significantly promote angiogenesis and diabetic wound healing as well as skin tissue formation. This bioactive biomaterial dressing (PABC) was based on the BGNC crosslinked double network hydrogel scaffold composed of polyethylene glycol diacrylate (PEGDA) and sodium alginate (ALG) which has shown wide applications in biomedicine. In the bioactive PABC hydrogel system, the ALG could crosslink with BGNC to form an antibacterial dynamic first network and the photocrosslinking of PEGDA was as the second network (Scheme 1). It is hypothesized that the bioactive PABC hydrogel scaffold dressing could efficiently seal off wound, absorb wound extravasate, resist bacterial infection, stimulate angiogenesis and accelerate healing of diabetic wound.

## Methods

### Synthesis and characterization of PABC hydrogel scaffold

The PAB (PABC) hydrogel scaffold was synthesized by UV light crosslinking of PEGDA in the presence of ALG and BGN (BGNC), and I2959 was used as a photoinitiator. PEGDA was dissolved in PBS solution at room temperature at a concentration of 8% (w/v). BGN (BGNC) was uniformly dispersed in PEGDA as solution A at different concentrations (0, 1, 2, 3 and 4 mg/mL). ALG was also dissolved in PBS (8 mg/mL), named solution B. Solution A and B were mixed in a volume ratio of 1:1 to form solution C. I2959 were added to solution C (0.5 wt% of the monomer). The PAB (PABC) hydrogel scaffolds were formed through crosslinking the solution C under a 365 nm UV light for 5 min. The PAB matrix was prepared without BGN under the same experimental conditions (PAB-0). The PAB scaffolds with 0 mg/mL, 1 mg/mL, 2 mg/mL, 3 mg/mL BGN were denoted as PAB-0, PAB-2, PAB-3 respectively. The PABC scaffolds with 3 mg/mL BGNC were denoted as PABC-3. To characterize the morphology and chemical compositions of the PAB composite scaffold, the surface field emission scanning electron microscopy (FESEM) and energy dispersive spectroscopy (EDS) images were collected on a Quanta 250 and an Oxford X-Max N, respectively. The chemical structure of PAB/PABC scaffold was tested by Fourier transform infrared spectroscopy (FT-IR, Nicolet 6700) from 4000-750 cm^-1^ with an average value of 32x scans.

### Multifunctional properties evaluations

The swelling behavior, rheological mechanical properties, self-healing ability, robust antibacterial activity of various scaffolds was evaluated according to previously reported methods 10. The details of evaluation procedures are given in the supporting information.

### Endothelial progenitor cells (EPCs) viability, proliferation, tube formation assessment

The EPCs derived from bone marrow of Sprague-Dawley (SD) rats were isolated and identified according to previous studies 34,38. The EPCs viability and proliferation was investigated through cell counting Kit-8 (CCK-8, Dojindo Co.) and Cell-Light™ EdU Apollo®567 *In Vitro* Kit (RiboBio Co., China) respectively. For tube formation assay, the cell suspension (5×10^3^ cells per well) pre-treated with PABC scaffold for 48 h was added into a µ-Slide (IBIDI, Germany) pre-coated with growth factor reduced basement membrane matrix (BD, Corning, US). After 6 h of incubation, the formed tubes were observed under a Nikon inverted light microscope. The number of tubes was counted according to the manufacturer's instructions. The particular steps and methods were described from the added files (supporting file).

### Animal experiment of diabetic full-thickness wounds

All animal protocols were approved by the Animal Care and Use Committee of Wenzhou Medical University. The male ICR mice with a weight of 30 to 35 g (SLAC laboratory animal company, China), were induced as the diabetic mice and used in this study. To induce a diabetic model, the mice were fasted overnight and intraperitoneally injected with 1% streptozotocin (STZ, 130 mg/kg) dissolved in 0.1 M sodium citrate buffer. After 2 w, the mice with a blood glucose level higher than 16.7 mM, together with the observed symptoms of weight loss and polyuria, were considered as diabetes. Two circular full-thickness wounds were created on the back of the mice with a diameter of 8 mm (to a level of panniculus muscle) and employed to evaluate wound healing ability of hydrogel scaffolds. The detailed procedure was shown in supported file.

### Evaluation of diabetic full-thickness wounds healing

At day 0, 7, 14 and 21, the wounds in different groups were recorded by a digital camera, and the wound margins were also traced. The wound healing rate can be calculated with the following equation: wound closure rates (%) = (A_0_-A_t_)×100%/A_0_, where A_0_ represented the wound area at day 0, and A_t_ is for the wound area at day 7, 14 and 21, respectively. The wound area was determined by the micrometer calipers. The blood flow in the diabetic wound was measured by a laser Doppler imager (MoorLDI-2, Moor Instruments Limited) and the supported file showed the method in detail. The wound healing process and performance was analyzed by the histological evaluation, immunofluorescence staining and western blotting analysis in which the detailed methods were seen in the supporting information.

### Statistical analysis

All data are presented as mean ± standard deviation (mean ± SD). The statistical differences were determined by the one-way analysis of variance (ANOVA) with GraphPad Prism 7.0 software and *p* < 0.05 was considered as statistical difference. The * *p* < 0.05 and ** p <0.01 were versus the indicated group.

## Results and Discussion

### Fabrication and characterizations of scaffold

Here, the double-network hydrogel was formed through the first photo-crosslinked PEGDA polymer network and the second ALG-BGNC network which was presented by the dynamic interaction of ALG and Ca^2+^/Cu^2+^ ions from BGN (BGNC) (Scheme 1A-B). The PAB (PABC) scaffold was obtained after irradiated for 5 min under a UV lamp (365 nm) with the presence of photoinitiator (I2959). The physicochemical evaluation of PAB scaffold with different proportions of BGN was performed by measuring the degradation, FTIR spectroscopy, swelling, and rheological behavior. It is hypothesized that the bioactive PABC hydrogel scaffold dressing could efficiently seal off wound, absorb wound extravasate, resist bacterial infection, stimulate angiogenesis and accelerate healing of diabetic wound (Scheme 1C).

**Figure 1:**
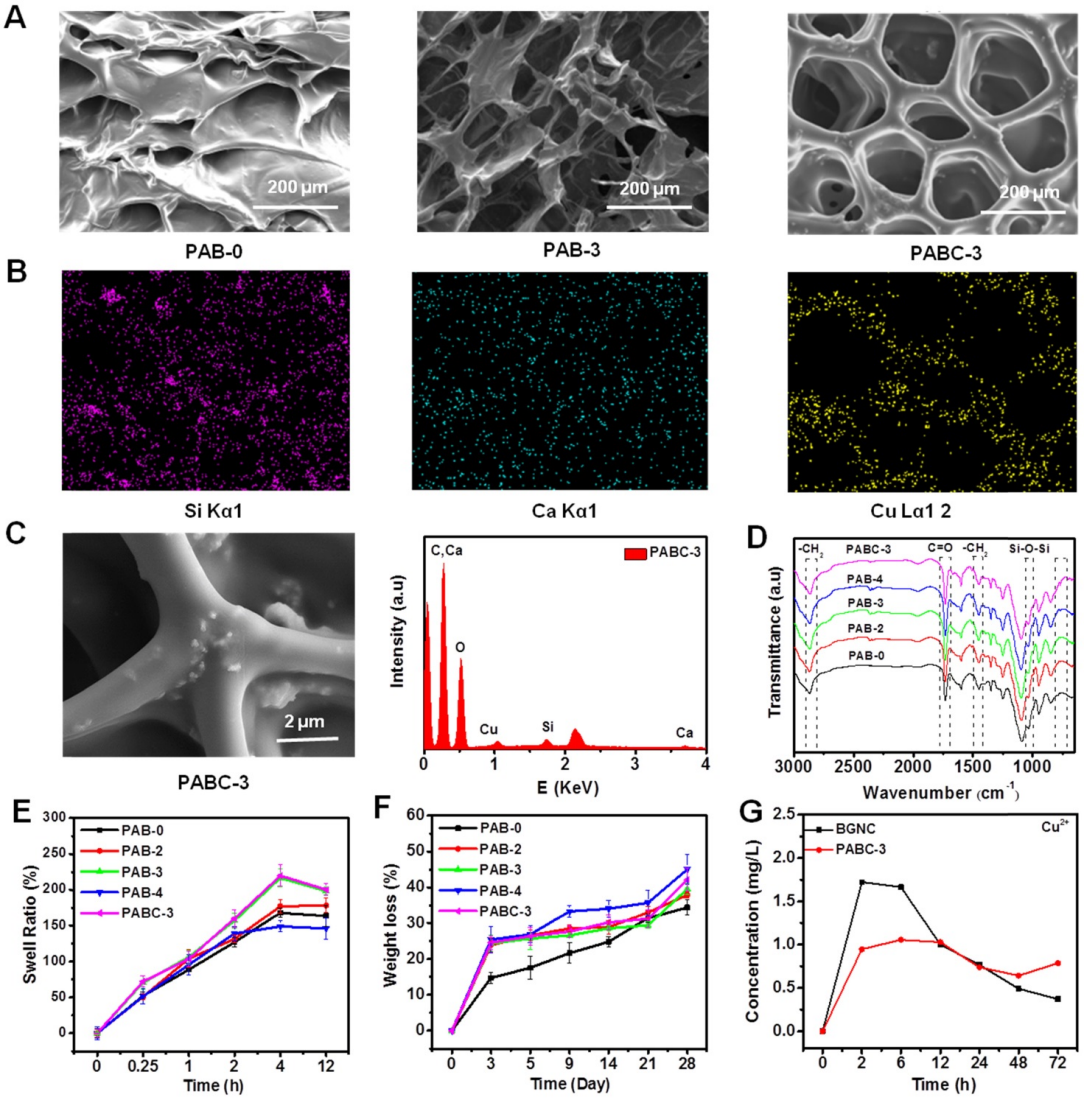
** Structure characterizations of PABC hydrogel. (A)** SEM images showing the porous structure of hydrogel; **(B)** Mapping pictures of each element (Si, Ca and Cu); **(C)** High magnification SEM image of PABC3 hydrogel and EDS spectra; **(D)** FTIR spectra between 3000-650 cm^-1^; **(E)** Swell ratio and **(F)** Weight loss of PABC hydrogels; **(G)** Cu^2+^ release behavior in BGNC and PABC hydrogel. (*p<0.05 and **p<0.01.)

The porous morphology of the PAB (PABC) scaffold was observed clearly through FESEM (Figure 1A). The uniform distribution of Si (green), Ca (blue), Cu (yellow) element in the hydrogel scaffolds could be clearly found from the EDS mapping (Figure 1B). The monodispersed BGNC could be seen in the wall of PABC scaffold (SEM image) and the EDS analysis also confirmed the elements composition of BGNC (Figure 1C). The FTIR spectra showed the chemical structure of various scaffolds (Figure 1D). All scaffolds exhibited the characteristic absorption bands of -CH_2_- at 2800-3000 and 1400-1500 cm^-1^ from PEGDA, C=O vibration absorption bands at 1730 cm^-1^ from ALG. After the addition of BGNs/BGNCs, the apparent Si-O-Si vibration absorption bands at 1035 cm^-1^ were observed clearly.

There was a significant effect for addition of BGNC on the swelling balance of PABC. The swelling ratio of the PAB/PABC scaffold increased almost linearly with the swelling time in the first 4 h and reached the swelling equilibrium after 12 h (Figure 1E). The equilibrium swelling ratios of PAB-0, PAB-2, PAB-3 and PABC-3 scaffold were 168.2%, 177.4%, 217.1% and 220.0%, respectively, suggesting that the addition of BGNCs significantly increased the swelling ability of PABC scaffold. This result could be attributed to the fact that the strong interaction between BGNCs and ALG probably decreased the crosslink density of PEGDA network. In addition to the swelling ratio, the addition of BGNs/BGNCs also improved the weight loss rate (degradation) of scaffold (Figure 1F). The results of ICP test of BGNC and PABC scaffold showed that the Cu^2+^ could be released sustainably with the soaking time (Figures 1G and Figure S1A-B). The incorporation of BGNCs could significantly retard the release rate of Cu^2+^ in scaffold. The sustained release of bioactive Cu^2+^ from PABC scaffold would probably enhance their bioactive functions including antibacterial ability and angiogenesis activity 35,36,39-41.

**Figure 2:**
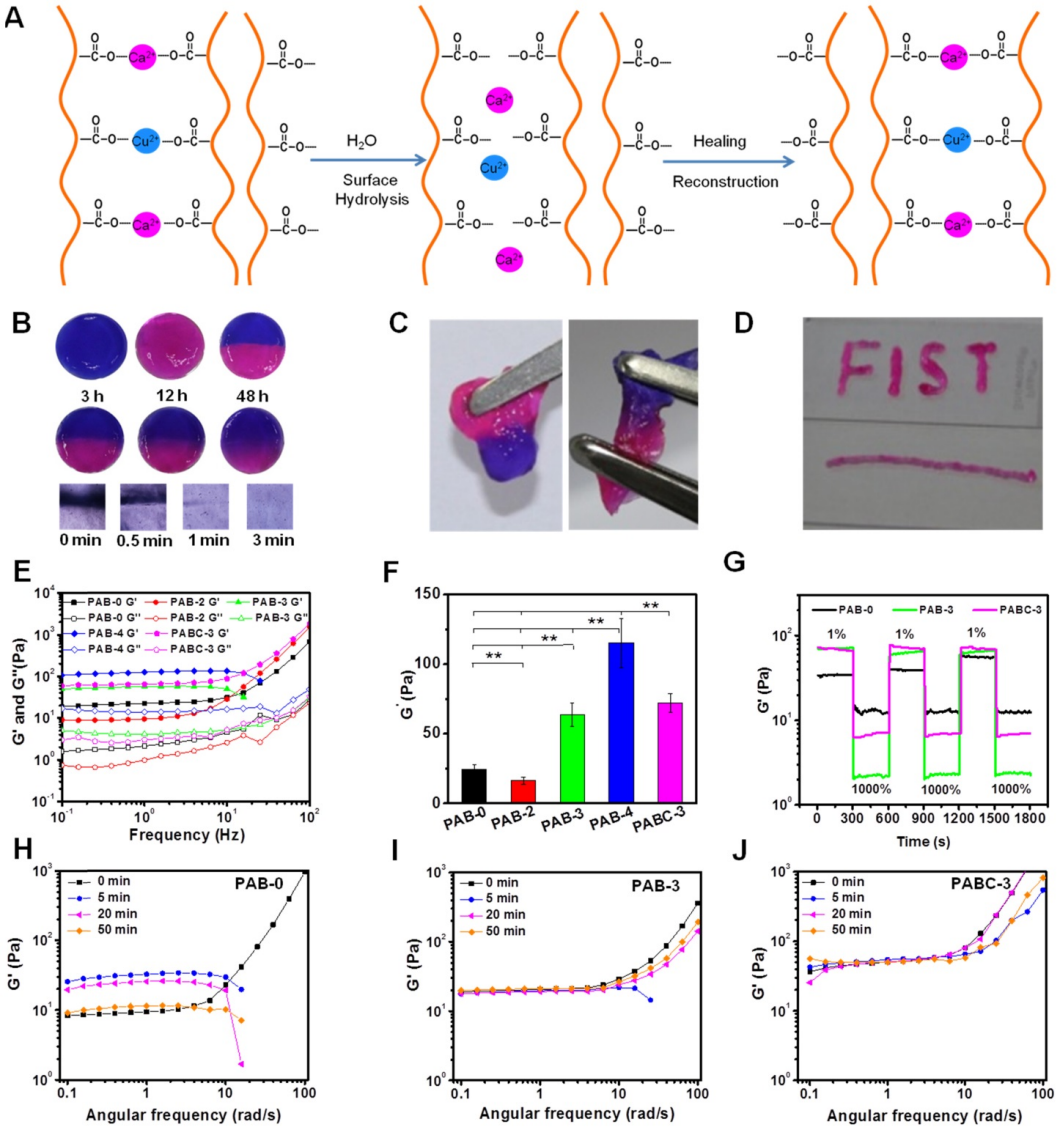
** Rheological mechanical properties of PABC hydrogel. (A)** Schematic of the self-healing mechanism for PABC hydrogel; **(B)** Self-healing process showed by optical and microscopic photos at different time points; **(C)** Optical photos of adhesive behavior after self-healing; **(D)** Optical photos of injectable behavior; **(E)** Storage moduli G' and loss moduli G” of PABC hydrogels with different frequencies; **(F)** Storage moduli G' of PABC hydrogels with different concentrations of BGN(C); **(G)** Storage moduli G' of PABC hydrogels by the continuous step strain (1% strain→1000% strain→1% strain) measurements to demonstrate the damage-healing property; **(H-J)** Storage moduli G' of (H) PAB-0, (I)PAB-3 and (J)PABC-3 hydrogels at different time points. (*p<0.05 and **p<0.01.)

The self-healing capacity, injectability and dynamic mechanical behavior of PAB/PABC scaffold were also investigated respectively. Previous study showed that the ionic bond could be rapidly formed between the carboxylic anion on the ALG chain and divalent metal ion in solution, which promoted the easy formation of the scaffold with dynamic reversible character 42 (Figure 2A). In this study, the Ca^2+^ and Cu^2+^ in BGN probably has the strong ionic bond interaction with ALG and this interaction could contribute to the self-healing ability of hydrogel (Figure 2A). When the separated two parts (blue and red) was put together, they recombined rapidly after 3 h (Figure 2B). After 12 and 48 h, the blue and red color was gradually fused together, suggesting the recovery of hydrogel. When gently pulling the self-healing PABC scaffold with tweezers, PABC scaffold exhibited excellent adhesive mechanical behavior (Figure 2C). The above experimental results confirmed that PABC scaffold exhibited good self-healing properties. Additionally, the PABC hydrogel scaffold could be easily extruded and form different letters, indicating the good injectable ability (Figure 2D). The rheological curves at different frequencies indicated that the storage and loss moduli (G', G”) of all hydrogel scaffolds exhibited similar nonlinear rheological behavior and increase with increasing shear rate (Figure 2E). It should be noted that there was a significant increase in the elastomeric storage modulus G' at 10 Hz as increasing BGN (Figure 2F). It was also observed that the modulus of hydrogel was a little decreased when low content of nanoparticles was added (PAB-2), which was probably due to the heterogeneous distribution of nanoparticles and decreased crosslinking density of polymer. As subjected to multiple high and low shear tests, it was found that after BGN's addition, the storage modulus G' of the PABC scaffold was more stable and the self-healing performance was better (Figure 2G and Figure S1). In order to further investigate the effect of the scaffold after self-healing, the storage modulus of scaffold after healing was tested at different time points (Figure 2H-J). There was no significant difference in the storage modulus for PAB-3 and PABC-3 scaffold at different time points, indicating the rapid recovery of scaffold after self-healing.

**Figure 3:**
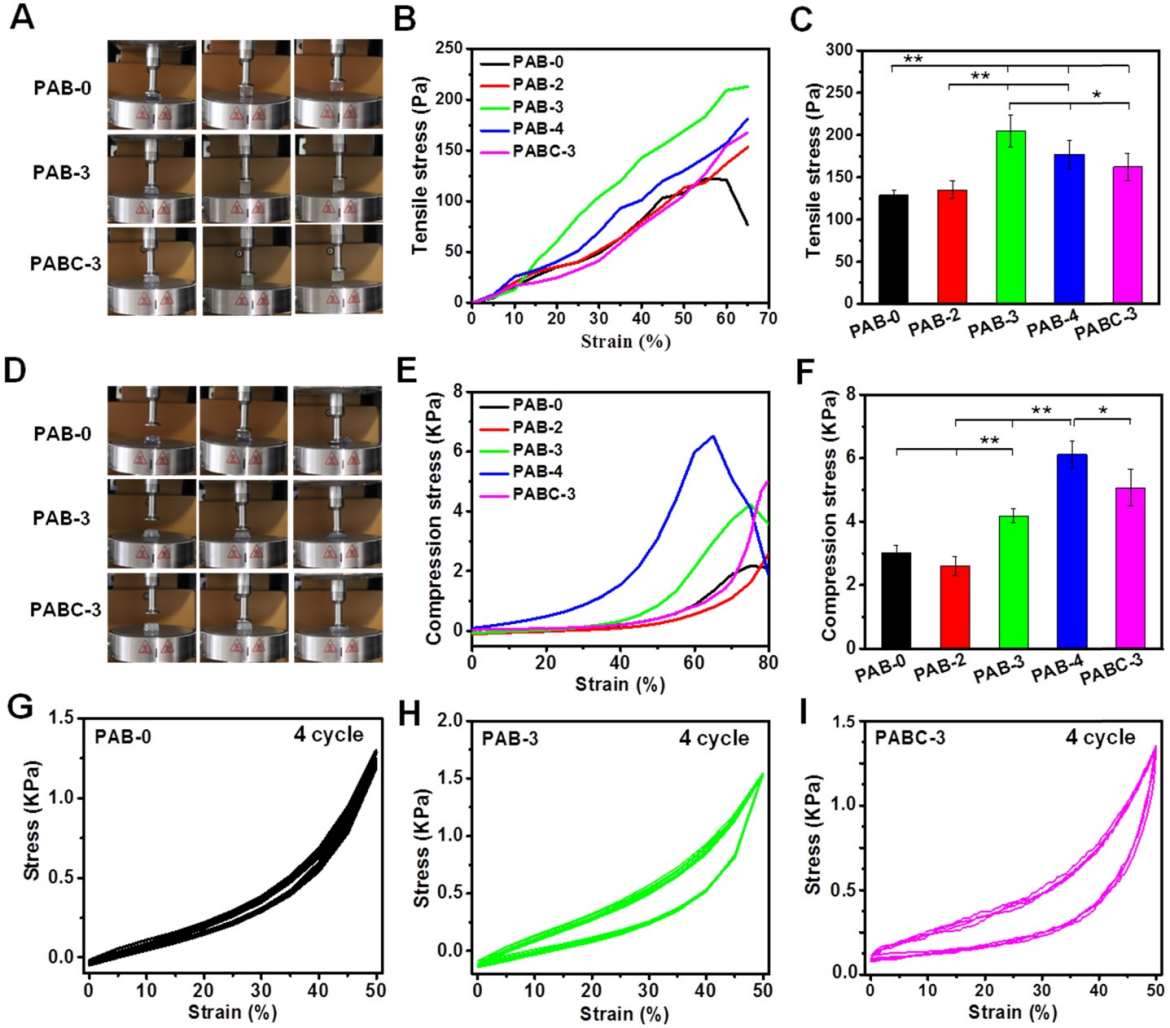
** Elastomeric mechanical properties of PABC hydrogels. (A)** Optical photos of tensile behavior; **(B)** Tensile stress-strain curves from 0% to 65% strain and **(C)** Tensile stress at 65% strain; **(D)** Optical photos of compressive behavior; **(E)** Compression stress-strain curves from 0% to 80% strain and **(F)** Compression stress at 80% strain; **(G-I)** Fatigue test after 4 cycles at 50% strain for (G) PAB-0, (H) PAB-3, (I) PABC-3 composite hydrogels. (*p<0.05 and **p<0.01.)

The tensile and compressive mechanical properties of PAB/PABC scaffold was also evaluated (Figure 3). PAB-based hybrid scaffold showed significantly high adhesive ability and tensile strength at a strain of 65% (Figure 3A-B). PAB-3 exhibited the best tensile strength of ~200 Pa (Figure 3C). The PAB/PABC scaffold also showed elastomeric compressive behavior (Figure 3D), and the addition of BGN significantly enhanced compressive strength of scaffold (Figure 3E). Compared with PAB-0 scaffold (~3 kPa), the PAB-3, PABC-3 and PAB-4 scaffold showed the significantly high compressive strength of ~4.2 kPa, 4.9 kPa and 6.1 kPa respectively (Figure 3F). Figure 3G-I show the antifatigue mechanical properties of scaffold after 4 cycles compressive test. The similar compress-release stress curves for PAB-0, PAB-3 and PABC-3 indicated their excellent elastomeric recovery ability. However, the hysteresis loops for PAB and PABC scaffold was significantly increased due to the addition of BGN/BGNC (Figure 3H-I). These results showed that the mechanical properties of hydrogel scaffolds were increased firstly and then decreased when the BGN contents increased. The previous studies showed the mechanical properties of nanocomposites were determined by the interaction between different phases 24,25. In our study, the hydrogel scaffolds with relative low concentration of BGN could efficiently increase the interaction between nanoparticle and ALG, but high content of BGN would aggregate in the hydrogel and decrease the tensile/compressive/elastomeric properties.

### Antibacterial activity evaluation

**Figure 4:**
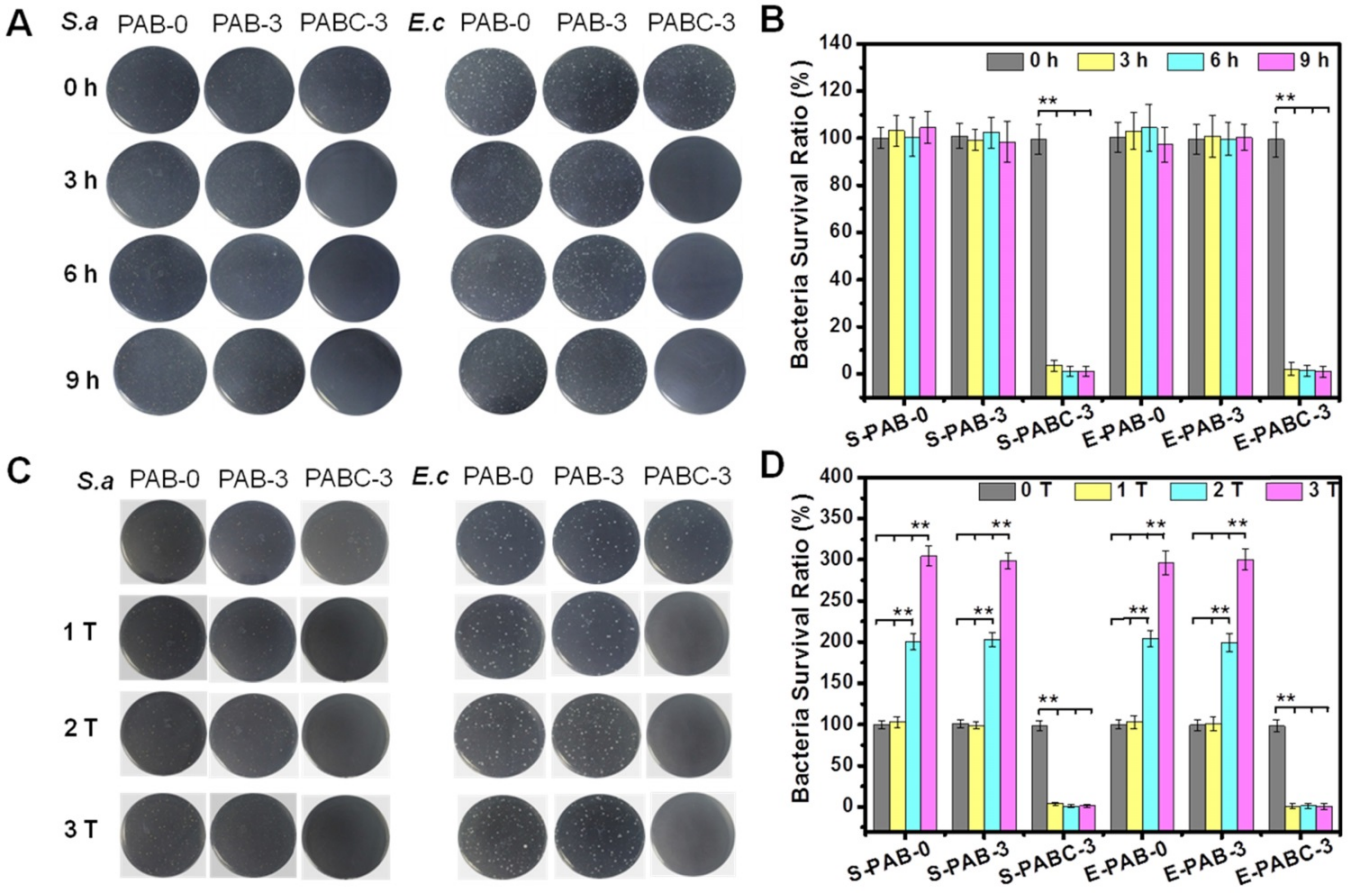
** Robust antibacterial activity of PABC hydrogel. (A-B)** Growth picture of bacteria (*S.aureus* and *E.coli*) on agar plate (A) and survival ratio (B) after co-culture with hydrogel for 0, 3, 6 and 9 h; **(C-D)** Bacteria (*S.aureus* and *E.coli*) growth graphs on agar plate (C) and survival ratio (D) after co-culture of hydrogel for repeatable times (adding bacteria respectively at 0, 3, 6 h). (*p<0.05 and **p<0.01.)

It was well known that Cu^2+^ with suitable concentration could eradicate common microorganisms, including bacteria, molds, fungi, and so on. Previous result showed that PABC scaffold demonstrated a sustained release behavior of Cu^2+^. Therefore, the possible antibacterial activity of PABC scaffold was investigated. Figure 4 shows the antibacterial activity of scaffold against *S.aureus* (Gram-positive bacteria) and *E.coli* (Gram-negative bacteria). The minimum inhibitory concentration (MIC) of PABC was 270 µg/mL for *S.aureus* and 125 µg/mL for *E.coli*, while the no obvious antibacterial activity of PAB was observed. Before incubation with scaffold (0 h), all groups showed high *S.aureus* and *E.coli* colonies (Figure 4A), as well as strong bacteria survival ratio (~100%) (Figure 4B). As compared to PAB-0 and PAB-3 scaffold, the number and ratio of surviving colonies of the two bacteria with PABC-3 scaffold was close to 0% after 3 h incubation (Figure 4A-B). Thus, PABC-3 scaffold had excellent antibacterial activity against *S.aureus* and *E.coli*. In order to further verify the robust antibacterial effect of PABC-3 scaffold, we investigated the recyclable antibacterial ability of PABC scaffold. The similar concentrations and volumes of bacteria to previous experiments were selected and added at 0, 3 and 6 h. For three consecutive additions of bacteria, the significantly high bacterial growth was found in PAB-0 and PAB-3 group, however, the survival rate of the bacteria remained less than 1% in PABC-3 group suggesting their excellent recyclable antibacterial effect (Figure 4C-D). The released Cu^2+^ in the PABC-3 scaffold was probably the main reason of the long-lasting antibacterial effect.

### *In vitro* endothelial cells biocompatibility and angiogenesis analysis

**Figure 5:**
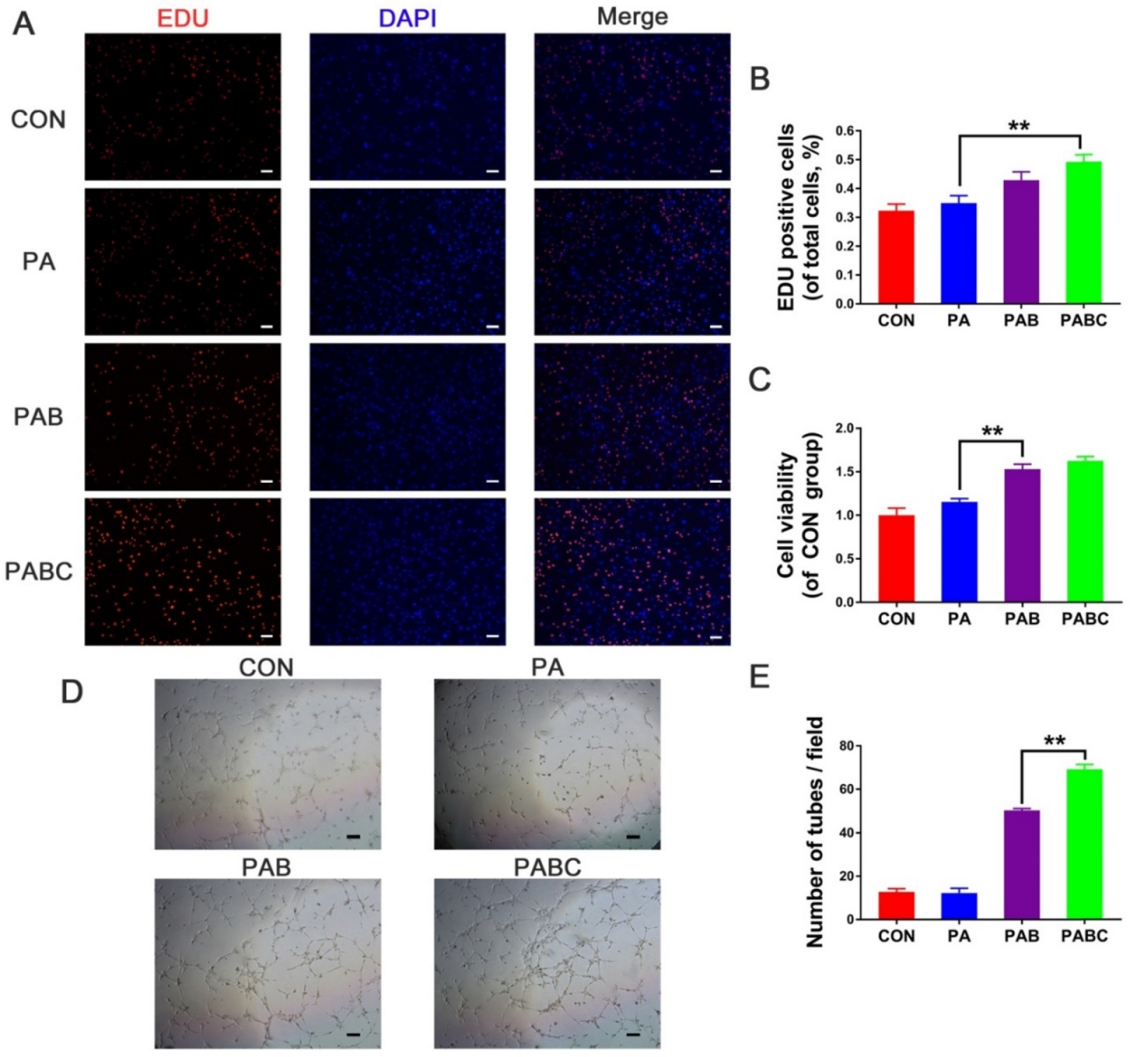
** Cell biocompatibility and *in vitro* angiogenesis of HUVECs stimulated for 48 h by hydrogels. (A-B)** Cell proliferation staining (A) and positive cells statistics (B) evaluation by EDU kit (scale bar 200 µm); **(C)** Cell viability test by CCK-8 kit; **(D-E)** Tube formation assay results including the phase contrast micrograph (D) and tube number analysis (E) (scale bar: 200 µm).

The effects of PABC scaffold on the cytotoxicity of EPCs were studied (Figure 5). EDU and CCK8 tests were used to assess the cell proliferation status influenced by the PA (PAB-0), PAB (PAB-3) and PABC (PABC-3) scaffold. After 48 h of co-culture with PABC scaffold, the cell viability and proliferation were significantly enhanced compared with PAB scaffold and control (Figure 5A-C). Although the EPCs proliferation of PAB scaffold group was also significantly higher than control, PABC group still had advantages on cell viability and proliferation with higher OD value of live cells and more EDU stained positive cells. *In vitro* angiogenesis was evaluated by the tube formation after EPCs were treated with scaffold for 48 h. It can be easily seen that PABC scaffold significantly enhanced the angiogenic ability of EPCs, and the significantly evidently higher number of newly-formed sprouting tubes were observed when compared with PA scaffold and control group (Figure 5D-E). Similar to the cell proliferation results, PAB scaffold also showed a positive effect on the tube formation of EPCs, indicating that the addition of bioactive glass could significantly enhance the angiogenic ability of PA matrix.

### Diabetic wound healing assessment

**Figure 6:**
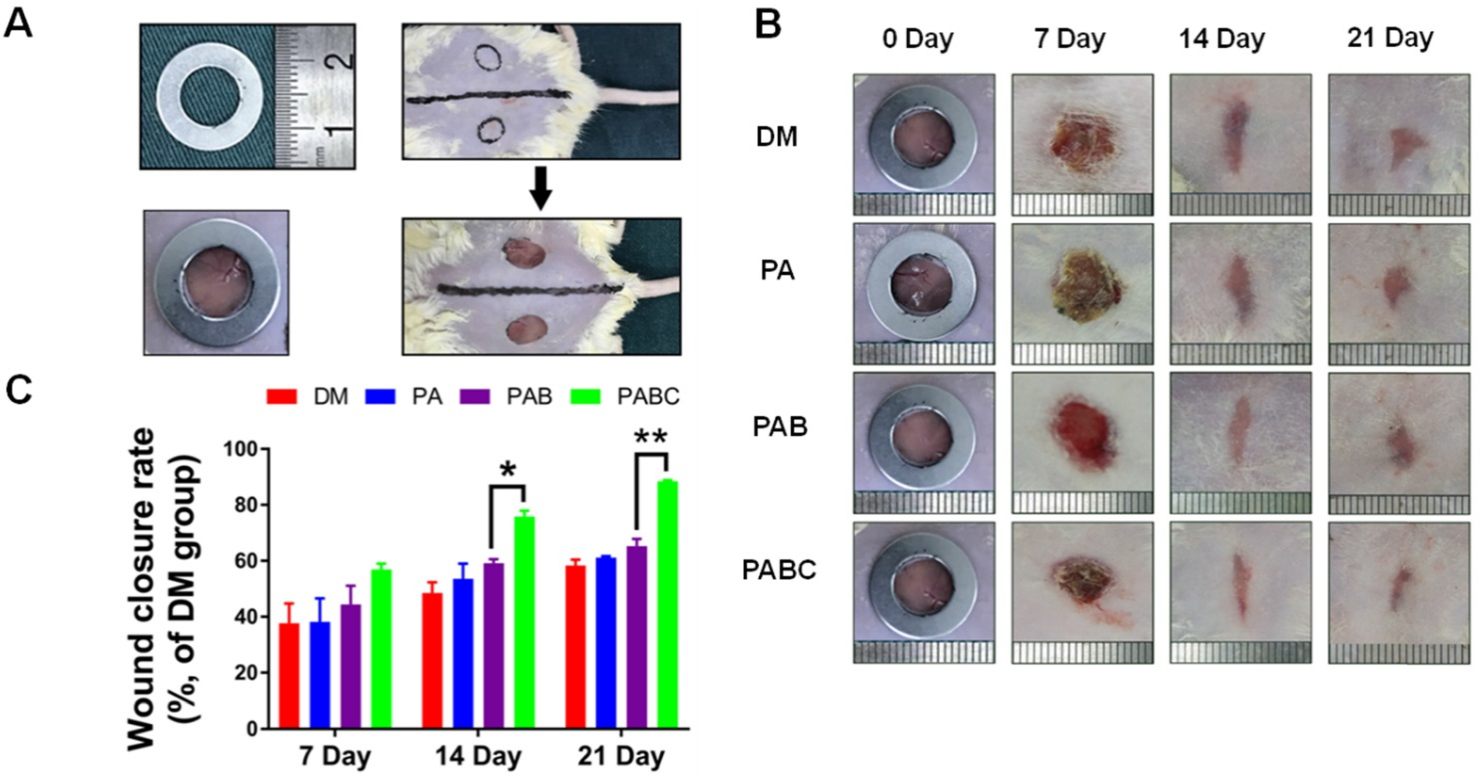
** Effect of hydrogel on diabetic wound healing. (A)** Construction of diabetic wound model in ICR mice (about 1 cm in diameter); **(B)** Gross observation of wound healing process during 21 days treatment by various hydrogels (PA, PAB, PABC), DM: Diabetes mellitus wound was used as a control; **(C)** Wound closure rates at day 7, 14 and 21. (*p<0.05 and **p<0.01.)

PABC and PAB scaffold possessed excellent cytocompatibility and promoted angiogenesis *in vitro*, indicating their potential application on diabetic wound healing. To investigate the healing effect of PABC scaffold on diabetic wounds, the as-prepared scaffold was used to treat the full-thickness cutaneous wounds of ICR mice (Figure 6A). Figure 6B shows the representative gross observation images of the diabetic wound healing process at different time points. The wounds treated by PABC scaffold healed much faster at the early stage of healing (day 7), and were basically covered with newly-formed epidermis at day 21. The statistical data also indicated that the wound healing rates at day 7, 14 and 21 were significantly higher in PABC group when compared with control and PA group, followed by the PAB scaffold group (Figure 6C).

**Figure 7:**
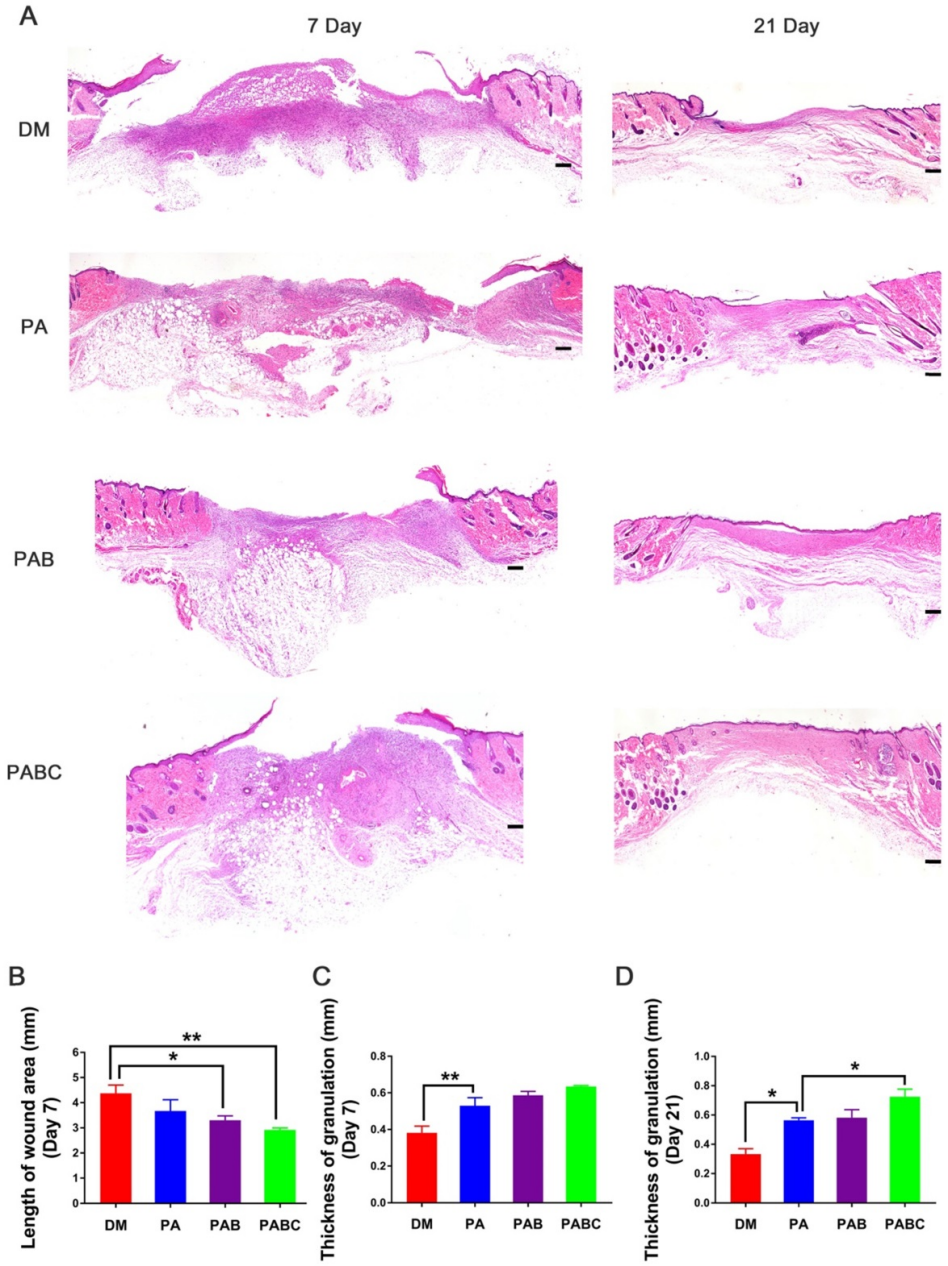
** Pathological evaluation of diabetic wound after treatment for 7 and 21 days. (A)** Representative H&E staining images at day 7 and 21; **(B)** Wound length analysis at day 7; **(C-D)** Quantification evaluation of granulation tissue thickness at day 7 (C) and day 21 (D). Scale bar: 200 µm. (*p<0.05 and **p<0.01.)

The healing pathology of wounds treated by injected sample was evaluated by H&E staining. The length of wound area was significantly shorter in PABC group than control at day 7, followed by PAB and PA groups (Figure 7A-B). The wounds treated with PABC and PAB scaffold were filled with abundant newly-formed granulation tissue with neo-epidermis, whereas less regenerated tissue was found in control and PA group (Figure 7A). The statistical data also confirmed that the thickness of granulation tissue was significantly higher in all scaffold groups when compared with control (Figure 7C). At day 21, the wounds were covered with neo-epidermis, and PABC group still showed the highest amount of granulation tissue (Figure 7D). Moreover, skin appendages-structure like tissue also appeared in PABC group, suggesting that the early fast healing in diabetic wounds would benefit the healing outcomes with less of scar tissue and skin appendages formation.

**Figure 8:**
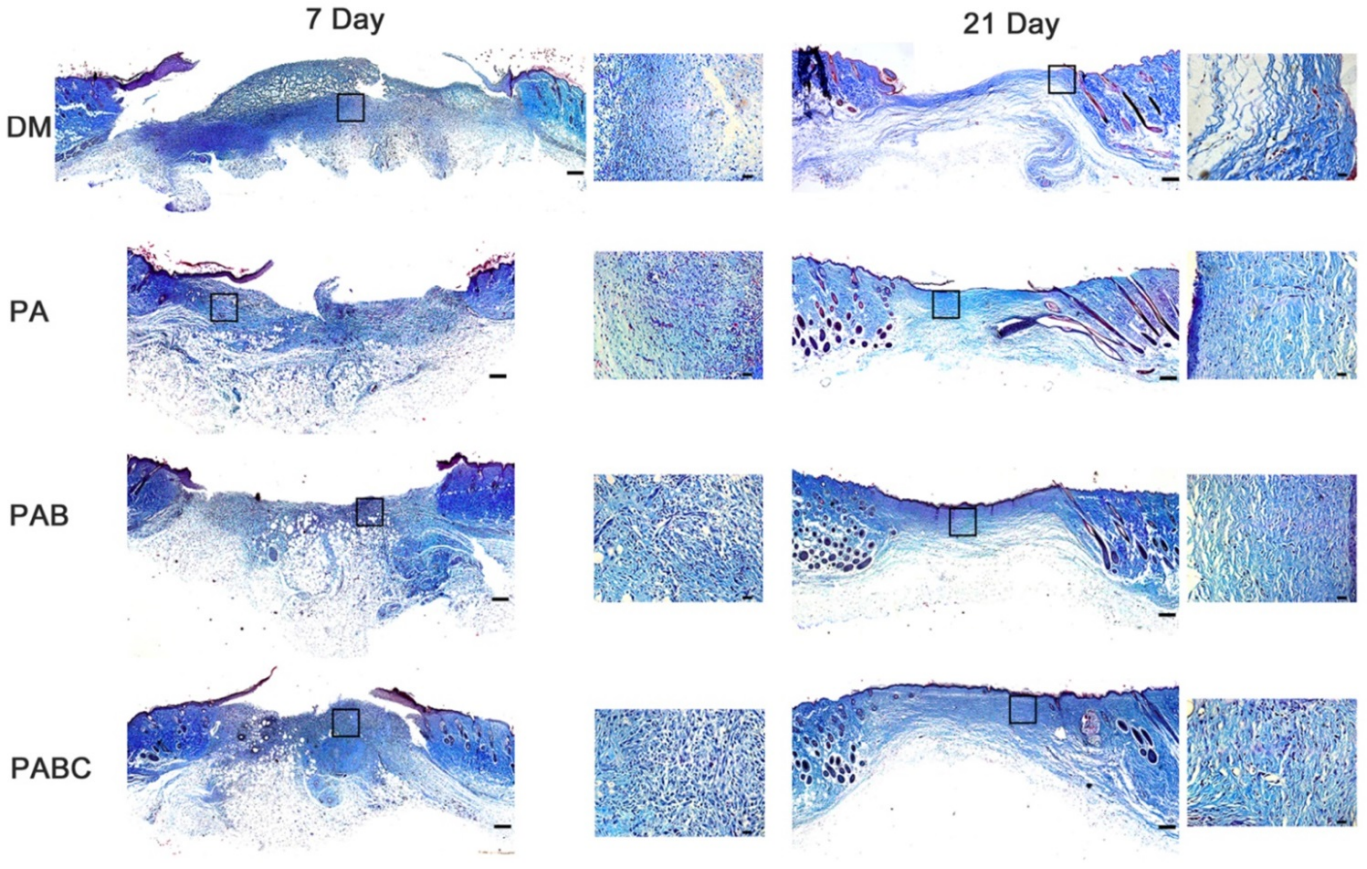
** Masson staining results of diabetic wound tissue after treatment of hydrogels at day 7 and 21.** Scale bar: 200 µm in original images and 20 µm in enlarged images.

Masson staining showed the collagen deposition and remodeling in diabetic wounds treated with PABC scaffold. The collagen amount in PABC group was obviously higher than other groups, which also confirmed the H&E staining results that PABC had the thickest granulation tissue (Figure 8). At day 21, the collagen fiber content was still higher in the scaffold treated wounds. Moreover, collagen fibers in PABC and PAB groups showed more organized structures with dense fiber density compared to control. These results indicated that PABC scaffold can accelerate the collagen deposition and remodeling in the diabetic wound healing process.

### Angiogenesis in diabetic wounds and related mechanism

**Figure 9:**
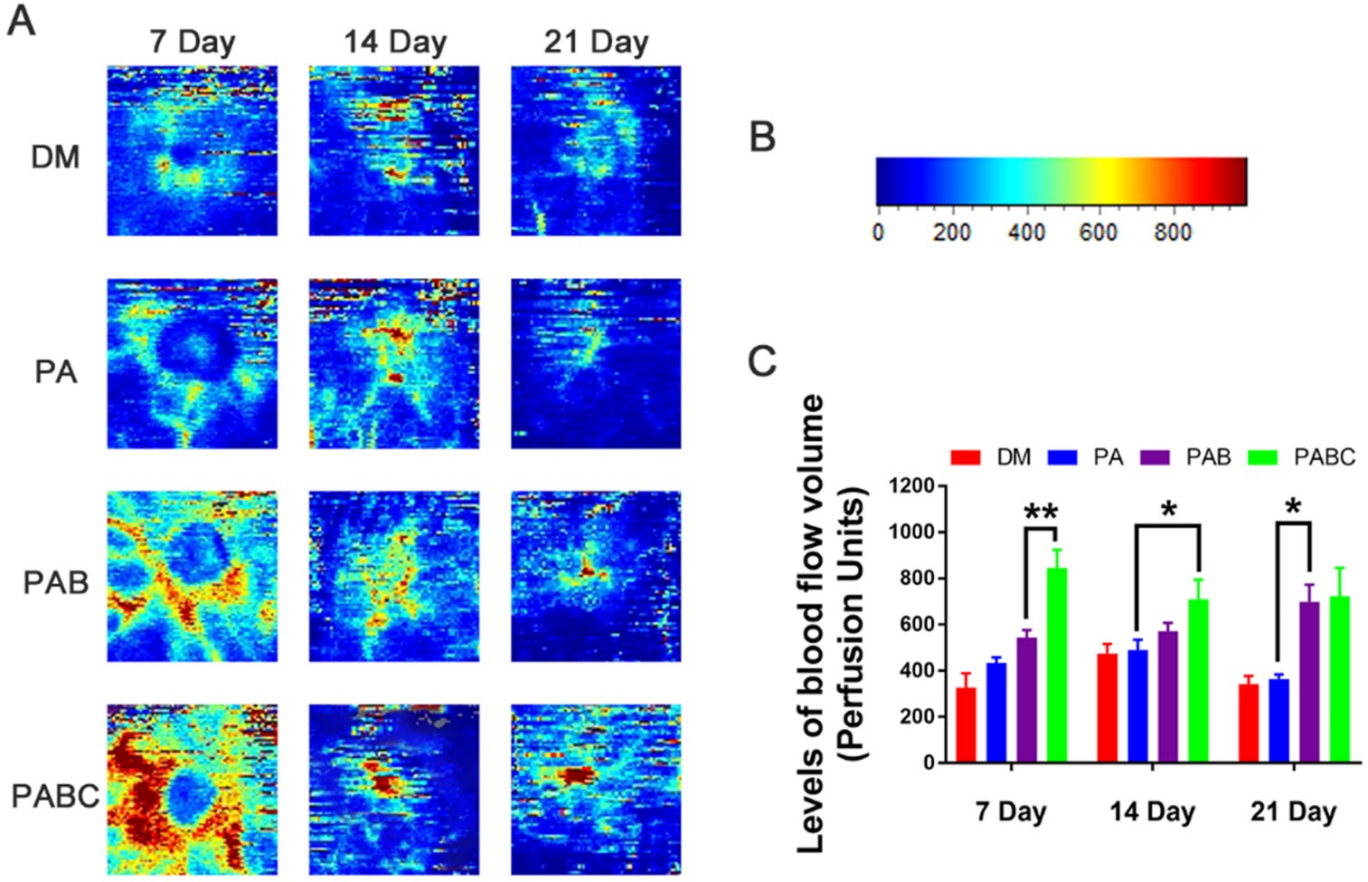
** Effect of hydrogel on functional blood vessel formation in diabetic wounds. (A)** Representative Laser Doppler scan images on the diabetic wound after treatment at day 7, 14, and 21; **(B)** Relative intensity bar of blood flow in wounds; **(C)** Quantification of blood flow volume at day 7, 14 and 21 using moorLDI Review V6.1 software. (*p<0.05 and **p<0.01.)

The above results showed that diabetic wound healing can be accelerated by the PABC scaffold. *In vitro* results also exhibited that PABC scaffold had potent pro-angiogenic effect on EPCs. The related mechanism about the enhanced *in vitro* angiogenesis performance for benefiting wound healing was then investigated. Laser Doppler analysis results showed that PABC group had the highest level of blood flow volume at all the three time points of healing, followed by the PAB group (Figure 9). The blood flow volume in PA group was compared to control, which both were significantly lower than PABC and PAB groups at day 7, 14 and 21 (Figure 9A). Additionally, the blood flow peaked at day 7, and then maintained at a relatively lower level in all groups (Figure 9B-C). The high blood perfusion in PABC scaffold means that more functional vessels with blood flow was achieved, which suggested that *in vivo* angiogenesis was also upregulated in diabetic wounds.

**Figure 10:**
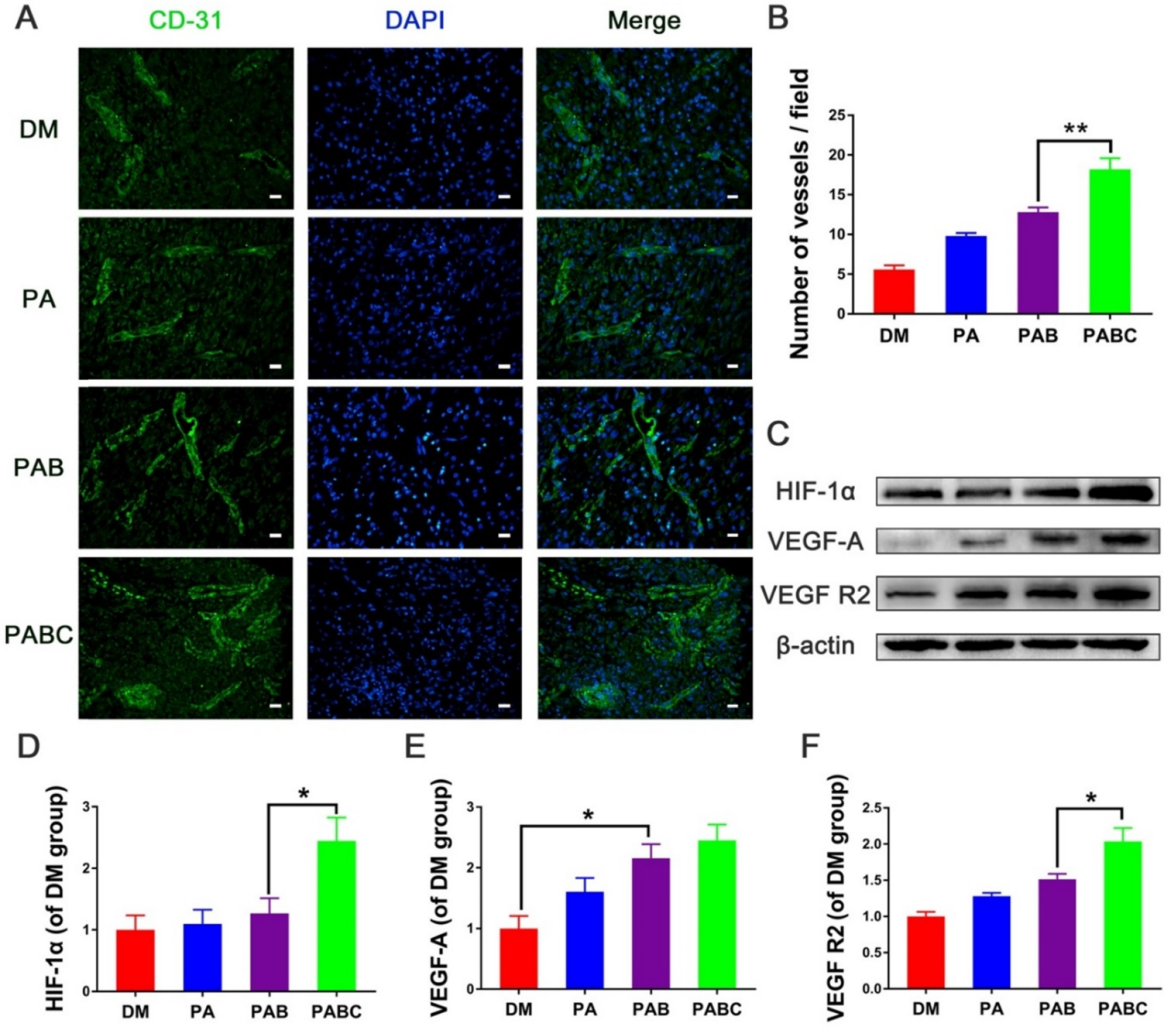
** Angiogenic proteins expression stimulated by hydrogel at day 7. (A)** Representative images of immunofluorescence staining of CD31 (scale bar: 20µm); **(B)** Quantification data of newly-formed vessels at day 7; **(C)** Western blood results of HIF-1α, VEGF-A, VEGF R2 in different groups; **(D-F)** quantification results of HIF-1α (D), VEGF-A (E), VEGF R2 (F) protein levels at different groups. (*p<0.05 and **p<0.01.)

**Figure 11:**
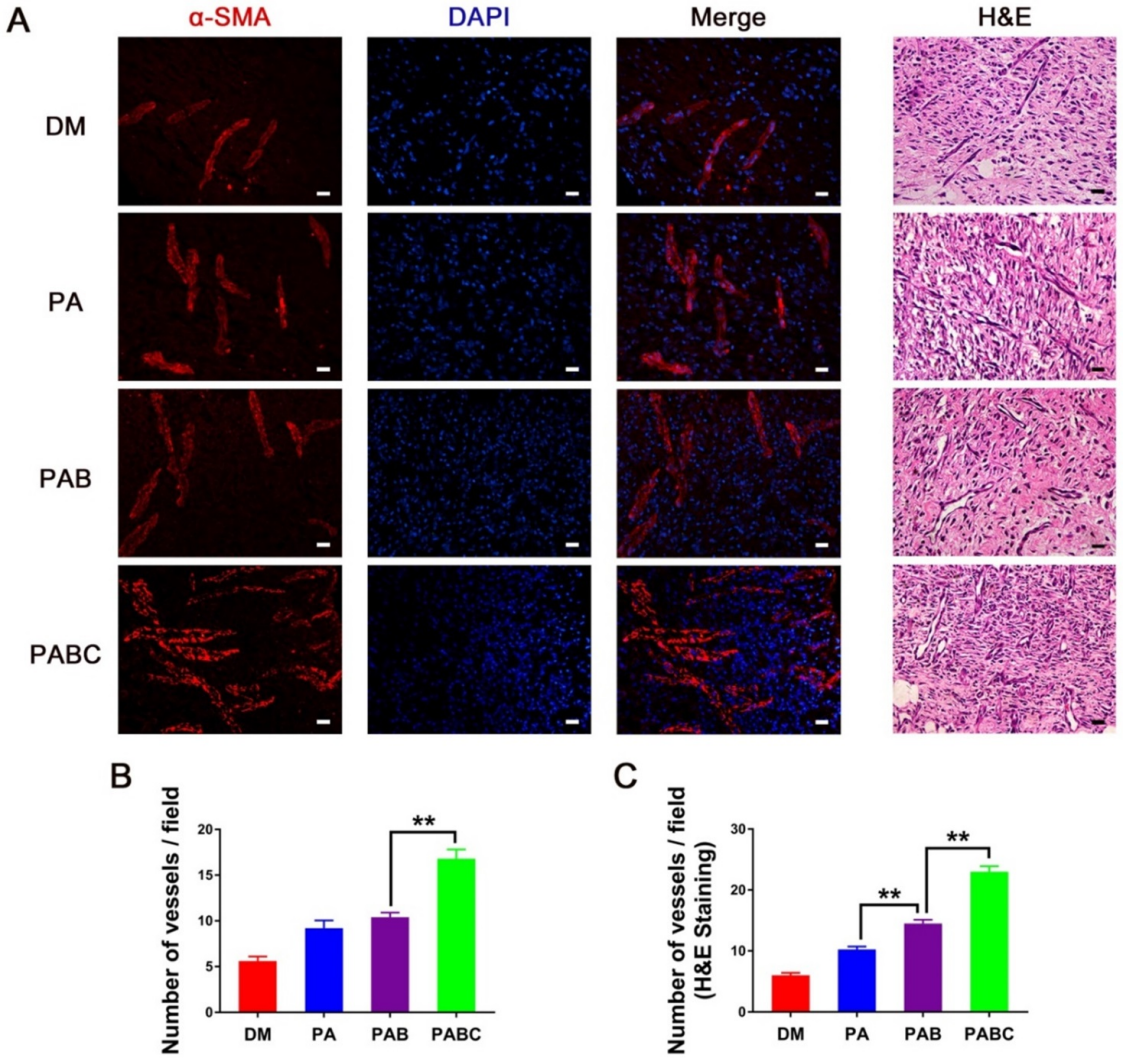
** Analysis of blood vessels formation in hydrogel treated wounds. (A)** Immunofluorescence and H&E staining results of α-SMA at day 7 (scale bar: 20 µm).; **(B-C)** Quantification evaluation of blood vessels at wound tissue of different groups, based on the α-SMA staining (B) and H&E staining (C). (*p<0.05 and **p<0.01.)

The CD31 and α-SMA immunofluorescence staining was then performed to indicate the newly-formed and relatively mature blood vessels stabilized with smooth muscle cells (SMCs) at day 7, respectively. The number of CD31 positive stained new blood vessels in PABC group was significantly higher than all other three groups, whereas the control wounds had very few vessels compared to others (Figure 10 A-B). To further study whether Cu^2+^ released from PABC hydrogel enhanced the angiogenesis of diabetic wounds, the related mechanism was also investigated. Figure 10C-F shows the expression of HIF-1α, VEGF-A, VEGF R2 in diabetic wounds. The band density of HIF-1α was significantly higher in PABC group, followed by PAB and PA group (Figure 10C-D). It should be mentioned that the protein level of VEGF-A, which almost had no expression in control wounds, was significantly up-regulated in PABC scaffold group (Figure 10E). The level of VEGF R2 showed a similar change pattern as HIF-1α, with increased expression in PABC group (Figure 10F). As for α-SMA staining, all wounds had positive staining at day 7 post treatment (Figure 11A). PABC scaffold group showed very strong positive staining of α-SMA with significantly higher number of vessels than PAB, PA and control group (Figure 11A-B). The representative images of H&E staining also confirmed that PABC group had the highest number of blood vessels (Figure 11C), which confirmed the immunostaining staining results of CD31 and α-SMA. The enhanced formation of blood vessels in diabetic wounds may be partially due to the incorporation of Cu^2+^ ions in the PABC hydrogel. The previous studies showed that the Cu^2+^ could stabilize HIF-1α and stimulate cells to secrete VEGF, therefore induce blood vessels formation and vascularization 39-41. In this study, the Cu^2+^ can be released from the PABC scaffold, which avoided the side-effect of burst release and may have a better bioactive function in stimulating angiogenesis. Taken together, PABC scaffold can efficiently promote the angiogenesis of diabetic wounds. The results indicated that the HIF-1α/VEGF/VEGFR2 could be enhanced by PABC scaffold, especially by the Cu^2+^ sustainably released from the scaffold during the healing process, and the up-regulated VEGF level plays a critical role in promoting the angiogenesis and neo-vascularization, further accelerating the diabetic wound repair and regeneration.

## Conclusions

In summary, we developed an injectable self-healing bioactive PABC hydrogel scaffold with robust antibacterial activity and angiogenesis capacity for treating diabetic wound. PABC scaffold exhibits excellent injectability, self-healing and viscoelastic mechanical properties, as well as repeatable antibacterial properties. PABC scaffold also has good cytocompatibility, significantly enhances the angiogenic ability of EPCs* in vitro*. PABC scaffold demonstrates a good adhesion on diabetic wound, accelerates collagen deposition and remodeling promotes the early angiogenesis/neovascularization through enhancing the HIF-1α/VEGF expression, and efficiently enhances diabetic wound healing. Moreover, PABC scaffold also enhances the skin appendages-like tissue formation, which suggests that our scaffold can probably benefit the skin tissue formation and decrease of scar tissue.

## References

[1] Nathan DM (2015). Diabetes: advances in diagnosis and treatment. Jama.

[2] Mudge EJ (2015). Recent accomplishments in wound healing. Int Wound J.

[3] Castleberry SA, Almquist BD, Li W, Reis T, Chow J, Mayner S (2016). Self-assembled wound dressings silence MMP-9 and improve diabetic wound healing in vivo. Adv Mater.

[4] Lim JZM, Ng NSL, Thomas C (2017). Prevention and treatment of diabetic foot ulcers. J Roy Soc Med.

[5] Laiva AL, O'Brien FJ, Keogh MB (2018). Innovations in gene and growth factor delivery systems for diabetic wound healing. J Tissue Eng Regen Med.

[6] Yan W, Liu H, Deng X, Jin Y, Wang N, Chu J (2018). Acellular dermal matrix scaffolds coated with connective tissue growth factor accelerate diabetic wound healing by increasing fibronectin through PKC signaling pathway. J Tissue Eng Regen Med.

[7] Xi Y, Ge J, Guo Y, Lei B, Ma P (2018). Biomimetic elastomeric polypeptide-based nanofibrous matrix for overcoming multidrug-resistant bacteria and enhancing full-thickness wound healing/skin regeneration. ACS Nano.

[8] Mir M, Ali MN, Barakullah A, Gulzar A, Arshad M, Fatima S (2018). Synthetic polymeric biomaterials for wound healing: a review. Prog Biomater.

[9] Xi Y, Ge J, Wang M, Chen M, Niu W, Cheng W, et (2020). al. Bioactive anti-inflammatory, antibacterial, antioxidative silicon-based nanofibrous dressing enables cutaneous tumor photothermo-chemo therapy and infection-induced wound healing. ACS Nano.

[10] Zhou L, Xi Y, Xue Y, Guo Y, Liu Y, Lei B (2019). Injectable self-healing antibacterial bioactive polypeptide-based hybrid nanosystems for efficiently treating multidrug resistant infection, skin-tumor therapy, and enhancing wound healing. Adv Funct Mater.

[11] Lee YH, Chang JJ, Chien CT, Yang MC, Chien HF (2012). Antioxidant sol-gel improves cutaneous wound healing in streptozotocin-induced diabetic rats. Exp Diabetes Res.

[12] Han L, Wang M, Li P, Gan D, Yan L, Xu J (2018). Mussel-inspired tissue-adhesive hydrogel based on the polydopamine-chondroitin sulfate complex for growth-factor-free cartilage regeneration. ACS Appl Mater Interfaces.

[13] Saldin LT, Cramer MC, Velankar SS, White LJ, Badylak SF (2017). Extracellular matrix hydrogels from decellularized tissues: Structure and function. Acta Biomater.

[14] Zhao X, Sun X, Yildirimer L, Lang Q, Lin Z, Zheng R (2017). Cell infiltrative hydrogel fibrous scaffolds for accelerated wound healing. Acta Biomater.

[15] Naahidi S, Jafari M, Logan M, Wang YJ, Yuan YF, Bae H (2017). Biocompatibility of hydrogel-based scaffolds for tissue engineering applications. Biotechnol Adv.

[16] Zhang YS, Khademhosseini A (2017). Advances in engineering hydrogels. Science.

[17] Carrejo NC, Moore AN, Silva TL, Leach DG, Li IC, Walker DR (2018). Multidomain peptide hydrogel accelerates healing of full-thickness wounds in diabetic mice. ACS Biomater Sci Eng.

[18] Elliott CG, Wang J, Walker JT, Michelsons S, Dunmore-Buyze J, Drangova M (2018). Periostin and CCN2 scaffolds promote the wound healing response in the skin of diabetic mice. Tissue Eng. Part A.

[19] Jeon EY, Choi BH, Jung D, Hwang BH, Cha HJ (2017). Natural healing-inspired collagen-targeting surgical protein glue for accelerated scarless skin regeneration. Biomaterials.

[20] Huang LC, Wang HC, Chen LH, Ho CY, Hsieh PH, Huang MY (2019). Bioinspired self-assembling peptide hydrogel with proteoglycan-assisted growth factor delivery for therapeutic angiogenesis. Theranostics.

[21] Zhang S, Liu Y, Zhang X, Zhu D, Qi X, Cao X (2018). Prostaglandin E2 hydrogel improves cutaneous wound healing via M2 macrophages polarization. Theranostics.

[22] Suhaeri M, Noh MH, Moon JH, Kim IG, Oh SJ, Ha SS (2018). Novel skin patch combining human fibroblast-derived matrix and ciprofloxacin for infected wound healing. Theranostics.

[23] Wang C, Wang M, Xu T, Zhang X, Lin C, Gao W (2019). Engineering bioactive self-healing antibacterial exosomes hydrogel for promoting chronic diabetic wound healing and complete skin regeneration. Theranostics.

[24] Xin TW, Gu Y, Cheng RY, Tang JC, Sun ZY, Cui W (2017). Inorganic Strengthened Hydrogel Membrane as Regenerative Periosteum. ACS Appl Mater Interfaces.

[25] Quinlan E, Partap S, Azevedo MM, Jell G, Stevens MM, O'Brien FJ (2015). Hypoxia-mimicking bioactive glass/collagen glycosaminoglycan composite scaffolds to enhance angiogenesis and bone repair. Biomaterials.

[26] Ojansivu M, Vanhatupa S, Bjorkvik L, Hakkanen H, Kellomaki M, Autio R (2015). Bioactive glass ions as strong enhancers of osteogenic differentiation in human adipose stem cells. Acta Biomater.

[27] Fiorilli S, Molino G, Pontremoli C, Iviglia G, Torre E, Cassinelli C (2018). The incorporation of strontium to improve bone-regeneration ability of mesoporous bioactive glasses. Materials.

[28] Shirazi AN, Fathi A, Suarez FG, Wang YW, Maitz PK, Dehghani F (2016). A novel strategy for softening gelatin-bioactive-glass hybrids. ACS Appl Mater Interfaces.

[29] Xue Y, Guo Y, Yu M, Wang M, Ma P, Lei B (2017). Monodispersed bioactive glass nanoclusters with ultralarge pores and intrinsic exceptionally high miRNA loading for efficiently enhancing bone regeneration. Adv Healthc Mater.

[30] Chen M, Zhao F, Li Y, Wang M, Chen X, Lei B (2020). 3D-printed photoluminescent bioactive scaffolds with biomimetic elastomeric surface for enhanced bone tissue engineering. Mater Sci Eng C.

[31] Xue Y, Niu W, Wang M, Chen M, Guo Y, Lei B (2020). Engineering a biodegradable multifunctional antibacterial bioactive nanosystem for enhancing tumor photothermo-chemotherapy and bone regeneration. ACS Nano.

[32] Li Y, Guo Y, Niu W, Chen M, Xue Y, Ge J (2018). Biodegradable multifunctional bioactive glass-based nanocomposite elastomers with controlled biomineralization activity, real-time bioimaging tracking, and decreased inflammatory response. ACS Appl Mater Interfaces.

[33] Gao W, Jin W, Li Y, Wan L, Wang C, Lin C (2017). A highly bioactive bone extracellular matrix-biomimetic nanofibrous system with rapid angiogenesis promotes diabetic wound healing. J Mater Chem B.

[34] Wang C, Wang Q, Gao W, Zhang Z, Lou Y, Jin H (2018). Highly efficient local delivery of endothelial progenitor cells significantly potentiates angiogenesis and full-thickness wound healing. Acta Biomater.

[35] Wang XJ, Cheng F, Liu J, Smatt JH, Gepperth D, Lastusaari M (2016). Biocomposites of copper-containing mesoporous bioactive glass and nanofibrillated cellulose: Biocompatibility and angiogenic promotion in chronic wound healing application. Acta Biomater.

[36] Das A, Sudhahar V, Chen G, Kim HW, Youn SW, Finney L (2016). Endothelial antioxidant-1: a key mediator of copper-dependent wound healing in vivo. Sci Rep.

[37] Xiao J, Zhu Y, Huddleston S, Li P, Xiao B, Farha OK (2018). Copper metal-organic framework nanoparticles stabilized with folic acid improve wound healing in diabetes. ACS Nano.

[38] Li Q, Tang G, Xue S, He X, Miao P, Li Y (2013). Silica-coated superparamagnetic iron oxide nanoparticles targeting of EPCs in ischemic brain injury. Biomaterials.

[39] Ryan EJ, Ryan AJ, Gonzalez-Vazquez A, Philippart A, Ciraldo FE, Hobbs C (2019). Collagen scaffolds functionalised with copper-eluting bioactive glass reduce infection and enhance osteogenesis and angiogenesis both in vitro and in vivo. Biomaterials.

[40] Yu Q, Han Y, Wang X, Qin C, Zhai D, Yi Z (2018). Copper silicate hollow microspheres-incorporated scaffolds for chemo-photothermal therapy of melanoma and tissue healing. ACS Nano.

[41] Xiao Y, Peng J, Liu Q, Chen L, Shi K, Han R (2020). Ultrasmall CuS@ BSA nanoparticles with mild photothermal conversion synergistically induce MSCs-differentiated fibroblast and improve skin regeneration. Theranostics.

[42] Lei Z, Wang Q, Sun S, Zhu W, Wu P (2017). A bioinspired mineral hydrogel as a self-healable, mechanically adaptable ionic skin for highly sensitive pressure sensing. Adv Mater.

