# Bioactive antibacterial silica-based nanocomposites hydrogel scaffolds with high angiogenesis for promoting diabetic wound healing and skin repair: Erratum

**DOI:** 10.7150/thno.73263

**Published:** 2022-05-28

**Authors:** Yannan Li, Tianzhen Xu, Zhuolong Tu, Wentong Dai, Yumeng Xue, Chengxuan Tang, Weiyang Gao, Cong Mao, Bo Lei, Cai Lin

**Affiliations:** 1 Department of Burn, the First Affiliated Hospital of Wenzhou Medical University, Wenzhou 325000, China; 2 Key Laboratory of Orthopedics of Zhejiang Province, the Second Affiliated Hospital and Yuying Children Hospital of Wenzhou Medical University, Wenzhou 325027, China; 3 School of Physical Science and Technology, Inner Mongolia University, Hohhot 010021,China; 4 Frontier Institute of Science and Technology, Xi'an Jiaotong University, Xi'an 710054, China; 5 Department of Orthopedics, Zhuji People's Hospital of Zhejiang Province, Shaoxing 312000, China

In our paper, Figure 4 and Figure 6 should be corrected as follows. The authors regret that this mistake was happened, although these mistakes have no effect on the conclusion in this paper.

## Figures and Tables

**Figure 4:**
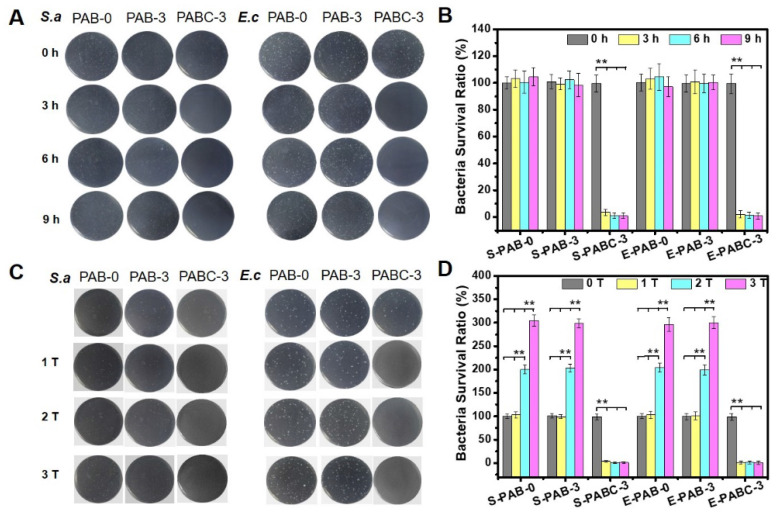
** Robust antibacterial activity of PABC hydrogel.** A-B) Growth picture of bacteria (*S.aureus* and *E.coli*) on agar plate (A) and survival ratio (B) after co-culture with hydrogel for 0, 3, 6 and 9 h; C-D) Bacteria (*S.aureus* and *E.coli*) growth graphs on agar plate (C) and survival ratio (D) after co-culture of hydrogel for repeatable times (adding bacteria respectively at 0, 3, 6 h). (*p<0.05 and **p<0.01.)

**Figure 6:**
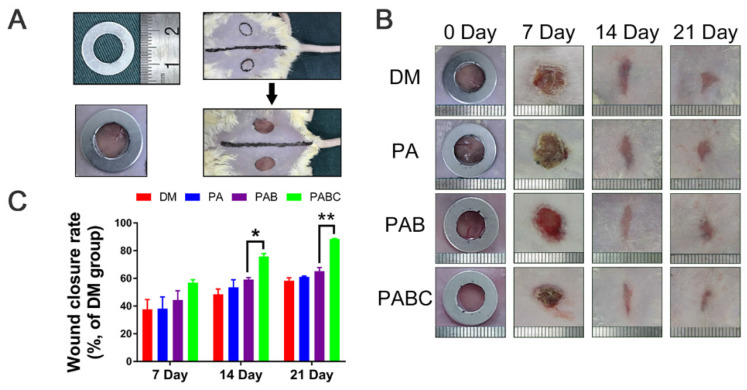
** Effect of hydrogel on diabetic wound healing.** (A) Construction of diabetic wound model in ICR mice (about 1 cm in diameter); B) Gross observation of wound healing process during 21 days treatment by various hydrogels (PA, PAB, PABC), DM: Diabetes mellitus wound was used as a control; (C) Wound closure rates at day 7, 14 and 21. (*p<0.05 and **p<0.01.)

