# Supplementary material for: Bioactive antibacterial silica-based nanocomposites hydrogel scaffolds with high angiogenesis for promoting diabetic wound healing and skin repair
Source: Theranostics. 2020 Mar 31;10(11):4929–43. doi: 10.7150/thno.41839 (PMC7163448; doi:10.7150/thno.41839)
Supplement: Supplementary file 1 — Supplementary figures and tables. [file thnov10p4929s1.pdf]

Supporting Information

Bioactive antibacterial silica-based nanocomposites hydrogel scaffolds with high angiogenesis for promoting diabetic wound healing and skin repair

Yannan Li [a, c #], Tianzhen Xu [b, e #], Zhuolong Tu [a], Wentong Dai [a], Yumeng Xue [d], Chengxuan Tang [b], Weiyang Gao [b], Cong Mao [b*], Bo Lei [d*], Cai Lin [a*]

[a] *Department of Burn, the First Affiliated Hospital of Wenzhou Medical University, Wenzhou 325000, China*

[b] *Key Laboratory of Orthopedics of Zhejiang Province, the Second Affiliated Hospital and Yuying Children Hospital of Wenzhou Medical University, Wenzhou 325027, China*

[c] *School of Physical Science and Technology, Inner Mongolia University, Hohhot 010021,China*

[d] *Frontier Institute of Science and Technology, Xi'an Jiaotong University, Xi'an 710054, China*

[e] *Department of Orthopedics, Zhuji People's Hospital of Zhejiang Province, Shaoxing 312000, China*

[#] *These authors contributed equally to this work.*

** Corresponding author:*

Dr. Cong Mao, Email: maocong@wmu.edu.cn

Prof. Bo Lei, Email: rayboo@xjtu.edu.cn

Prof. Cai Lin, Email: lincai0577@hotmail.com

Experimental section

1. Materials

Polyethylene glycol diacrylate (PEGDA-700, 98%), alginate sodium (ALG, 99%), 2-hydroxy-4'-(2-hydroxyethoxy)-2-methylpropiophenone (I 2959, 98%), dodecylamine (DDA, 99%), ethyl alcohol (ETOH, 99%), tetraethyl orthosilicate (TEOS, 99%), triethylphosphate (TEP, 99%), calcium nitrate tetrahydrate (CaN, 99%) cupric nitrate trihydrate (CuN, 99%) and phosphate buffered saline (PBS, 99%) , Luria-Bertani Agar (LBA), Mueller-Hinton Broth medium (MHB) were purchased from Aldrich. Deionized water was used for all experiments.

2. Synthesis of bioactive glass nanoparticles (BGN) and bioactive glass nanoparticles with copper (BGNC).

BGN was prepared by a traditional template method in our previous study, in which DDA was a catalyst and a template agent. In brief, DDA was dissolved in ethanol and deionized water with a volume ratio of 1:1, and TEOS, TEP, and CaN were added sequentially. After the reaction, the product was centrifuged, lyophilized and calcined in a muffle furnace to get the final product. The synthesis method of BGNC was basically the same as the above, in which the original CaN was replaced with the CaN and CuN mixture (the molar ratio of CaN and CuN = 1:1).

3. Swelling behavior

To study the swelling behavior, PAB composite hydrogels were tested by a gravimetric method. Briefly, the PAB composite hydrogel was immersed in deionized water at room temperature. Samples were taken from the water at selected different time points and surface droplets were removed. Then, the sample was weighed and placed back in deionized water. Swelling ratio Q_w is calculated as follows:

$$Q_w = \frac{W_t - W_0}{W_0} \times 100\%; \quad (1)$$

where W_0, W_t were the initial weight of the sample, and the weight of the sample after swelling at a certain time point, respectively.

4. Rheological and self-healing studies

Rheological measurements of PAB hydrogels were performed with a rheometer (DHR-2, TA) at 25°C. Using a parallel plate with a diameter of 20 mm, the shear frequency was increased from 0.1 to 100 Hz for stable shear rheological measurements. At different shear strains (1%-1000%), the change in storage modulus and loss modulus of different samples was measured. After the hydrogels were completely damaged, the storage modulus and loss modulus of the PAB hydrogel at different shear strains were recorded over time.

5. Mechanical properties Test

In this work, the mechanical test was mainly evaluated from the tension and compression of the material by the rheometer. The detailed method was as follows. Firstly, a cylindrical hydrogel was prepared with a 20 mm diameter (the same size as a parallel plate) and a 10 mm thickness. Then, the hydrogel was vertically placed and completely in contact between the operator station and the parallel plate. At 25°C, records were collected by the rheometer for the changes in the material surface tension (the parallel plate moved up) or compression (the parallel plate moved down) with a constant rate of movement (100 μm/s).

6. Anti-bacterial activity

To evaluate the anti-bacterial activity of hydrogels, Gram-negative bacteria (*Escherichia coli*, *E.coli*) and Gram-positive bacteria (*Staphylococcus aureus*, *S. aureus*) were selected for the experiment. First, a cylindrical hydrogel (0.8 mL) was prepared in a centrifuge tube and sterilized

at 55°C. Bacteria, grown in approximately 10^8 cells/mL of MHB broth were centrifuged out of the MHB broth, washed and diluted 100-fold in a sterile PBS solution. Then, the hydrogel was immersed in the 8 mL bacterial suspension. After incubation at 37 °C for 3 h, 6 h and 9 h on the shaker, the original solution was diluted to 100-fold and 10 μL of solution were prepared LBA plates to determine the amount of residual bacteria. Another option was to add the same volume of broth residually for 0 h, 3 h and 6 h. Then, after 3 h, 6 h, and 9 h, the original bacterial solution was diluted 100-fold and 10 μL of solution were prepared LBA plates to determine the amount of bacteria left PAB-0 and PAB-3 were the control group, and PABC-3 was the experimental group.

To test the minimum inhibitory concentration (MIC), PAB and PABC were evenly dispersed in PBS (4 mg/mL) to add to the 1st well , and diluted into the 2nd to 10th well plate in a doubling manner. Equal amounts of bacteria solution were added to the 1st to 11th wells, and sterile culture solution was added to the 12th well as a blank control. The 96-well plate with the uniform solutions was placed in a 37 °C microbial incubator for 8-10 h. Finally, we counted the number of transparent wells in the plate and calculated the minimum inhibitory concentration (MIC).

7. *In vitro* assessment of PABC hydrogel on endothelial progenitor cells (EPCs)

Endothelial progenitor cells (EPCs) derived from bone marrow of Sprague-Dawley (SD) rats were isolated and identified according to previous studies [1, 2]. All EPCs of passages 2-4 used in this study were cultured in EGM-2MV SingleQuots medium (Lonza, MD, USA). The cell viability was evaluated by Cell Counting Kit-8 (CCK-8, Dojindo Co, Japan). Briefly, cells were incubated with 100 μL PABC hydrogel in a 6-well plate for 48 h. After that, 5×10^3 cells were transferred into a 96-well plate and cultured with fresh medium for another 24 h. 10% CCK-8 solution supplemented with culture medium was then added into the each well and cultured for

another 2 h. The absorbance of 450 nm was measured by a microplate reader (Multiskan GO; Thermo Scientific).

Cell proliferation was tested through an EdU test using a Cell-Light™ EdU Apollo®567 *In Vitro* Kit (RiboBio Co., China) based on the manufactures' instruction. Briefly, EPCs were cultured with PABC hydrogel for 48 h. EDU solution was then added into each well containing 1x10^5 cells and incubated for 6 h. After that, cells were fixed with 4% paraformaldehyde and incubated with 2 mg/mL aminoacetic acid for 5 min under oscillation, followed by 0.5% TritonX-100 PBS treatment. Next, Apollo reagent was added into the culture plate and incubated for 1 h. Cells were then treated with PBS containing 0.5% TritonX-100, and methanol, respectively. Hoechst 33342 solution was then used to stain the cell nuclei. The stained cells were observed under an inverted fluorescent microscope (Nikon).

8. Preparation of diabetic wound and implantation of hydrogels

After another week of observation, the mice were anesthetized with 4% chloral hydrate (150 mg/kg), shaved and sterilized. Sequentially, two circular full-thickness wounds were created on the back of the mice with a diameter of 8 mm (to a level of panniculus muscle). After saline irrigation, the prepared PA, PAB and PABC scaffolds was then used to cover the wounds. Saline treatment was used as control. All mice were kept individually in a specific pathogen free (SPF) animal room and fed with standard food and water with close observation.

9. Blood flow measurement

At day 7, 14 and 21, mice were anesthetized, shaved and the blood flow around the wound area was assessed by a laser Doppler imager (MoorLDI-2, Moor Instruments Limited, Devon, UK). The scanning parameters were set with a laser wavelength of 633 nm, scan distance of 55 cm

and duration of 5 min. The obtained data was then analyzed by the MoorLDI Review V6.1 software.

10. Histological evaluation, immunofluorescence staining and western blotting analysis

Wound samples were collected at day 7, 14 and 21, fixed in 4% paraformaldehyde, dehydrated and embedded in paraffin. The samples were then cut into 5 μm thick sections and mounted on slides for staining. Hematoxylin and Eosin (H&E) (Beyotime Institute of Biotechnology, China) staining and Masson trichrome staining (Beyotime Institute of Biotechnology, China) were performed according to the manufactures' instructions. After staining, images were taken under an Olympus (IX 71) microscope. The thickness of granulation tissue in wound healing was analyzed based on the sections of H&E and Masson staining.

To evaluate the effect of PABC scaffold on the angiogenesis of regenerated tissue, immunofluorescence staining of CD31 (endothelial cells) and α-smooth muscle actin (α-SMA) on wound sections was performed at day 7. Briefly, the sections were deparaffinized and rehydrated, blocked with 5% bovine serum albumin (BSA) (Beyotime) and incubated with the primary antibodies overnight at 4 °C. Sequentially, slides were treated with secondary antibodies and nuclei were then stained by DAPI. All slides were then observed under a fluorescence microscope (Zeiss 40FL Axioskop) and the number of vessels was also counted. The primary antibodies used here were α-SMA rabbit polyclonal antibody (Proteintech Group, USA) and anti-CD31 antibody (P2B1) ab24590 (Abcam, Cambridge, UK) and Alexa Fluor® 647 or 488 affiniPure goat anti-rabbit IgG (H+L) (Yeasen, Shanghai, China) was used as a secondary antibody.

After 7-day of treatment by PABC scaffold, wound samples were collected and homogenized in RIPA lysis buffer. The obtained protein extracts were then quantified with a BCA reagent. 65 μg

tissue proteins were loaded with loading buffer and heated at 95 °C for 5 min. Then proteins were separated by 12% SDS-PAGE and transferred to PVDF membranes. The membranes were then blocked by 5% non-fat milk for two hours and incubated with primary antibodies overnight at 4 °C, followed by incubation with horseradish peroxidase-conjugated secondary antibodies for 2 h at room temperature. The bands were visualized by an electrochemiluminescence reagent (Invitrogen) and imaged under a ChemiDocXRS+ Imaging System (Bio-Rad). The primary antibodies used here include anti-HIF-1α (1:500, Abcam, Cambridge, UK), anti-VEGF (1:1000, Proteintech Group, USA), and anti-VEGF R2 (1:1000, Proteintech Group, USA).

Figure captions

Figure S1. Structure characterizations of PABC hydrogel. (A) Si release behavior in BGNC and PABC hydrogel, (B) Ca^{2+} release behavior in BGNC and PABC hydrogel.

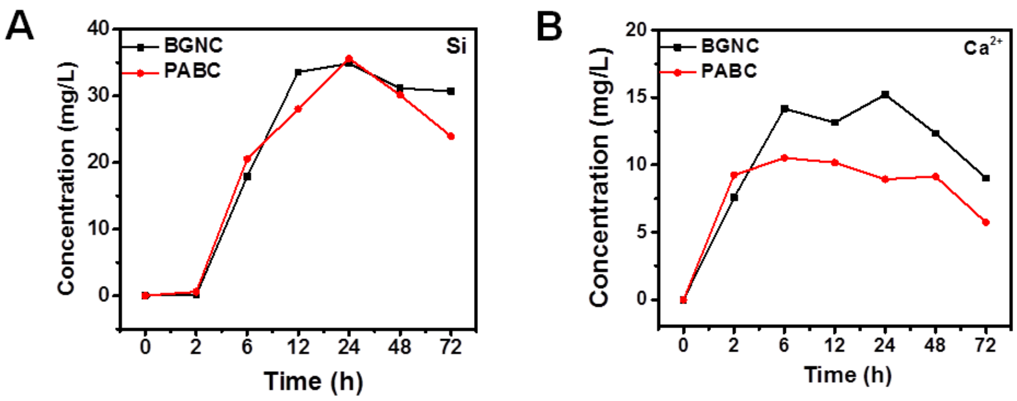

Figure S1
